# Dynamic compression of water to conditions in ice giant interiors

**DOI:** 10.1038/s41598-021-04687-6

**Published:** 2022-01-13

**Authors:** A. E. Gleason, D. R. Rittman, C. A. Bolme, E. Galtier, H. J. Lee, E. Granados, S. Ali, A. Lazicki, D. Swift, P. Celliers, B. Militzer, S. Stanley, W. L. Mao

**Affiliations:** 1 Fundamental Physics Directorate, SLAC National Accelerator Laboratory, Menlo Park, CA 94025 USA; 2 Geological Sciences, Stanford University, Stanford, CA 94305 USA; 3 Shock and Detonation Physics, Los Alamos National Laboratory, Los Alamos, NM 87545 USA; 4 Linac Coherent Light Source, SLAC National Accelerator Laboratory, Menlo Park, CA 94025 USA; 5 Shock Physics, Lawrence Livermore National Laboratory, Livermore, CA 94550 USA; 6 Earth and Planetary Science, University of California, Berkeley, CA 94720 USA; 7 Earth and Planetary Sciences, Johns Hopkins University, Baltimore, MD 21218 USA; 8 Applied Physics Lab, Johns Hopkins University, Laurel, MD 20723 USA

**Keywords:** Giant planets, Structure of solids and liquids

## Abstract

Recent discoveries of water-rich Neptune-like exoplanets require a more detailed understanding of the phase diagram of H_2_O at pressure–temperature conditions relevant to their planetary interiors. The unusual non-dipolar magnetic fields of ice giant planets, produced by convecting liquid ionic water, are influenced by exotic high-pressure states of H_2_O—yet the structure of ice in this state is challenging to determine experimentally. Here we present X-ray diffraction evidence of a body-centered cubic (BCC) structured H_2_O ice at 200 GPa and ~ 5000 K, deemed ice XIX, using the X-ray Free Electron Laser of the Linac Coherent Light Source to probe the structure of the oxygen sub-lattice during dynamic compression. Although several cubic or orthorhombic structures have been predicted to be the stable structure at these conditions, we show this BCC ice phase is stable to multi-Mbar pressures and temperatures near the melt boundary. This suggests variable and increased electrical conductivity to greater depths in ice giant planets that may promote the generation of multipolar magnetic fields.

## Introduction

Understanding the phase diagram of H_2_O, a ubiquitous molecule in the Universe and a primary building block of volatile-rich giant planets, is of crucial importance for condensed matter physics, solid-state chemistry, and planetary science. During the formation of Uranus and Neptune in the outer region of our solar system, massive amounts of H_2_O were accreted and are now stored at hundreds of GPa pressures in their interiors^1^. During the visit of the Voyager II spacecraft, its magnetometer revealed surprising non-axisymmetric, non-dipolar magnetic fields for the ice giants that differed substantially from the strong dipolar fields of Jupiter and Saturn^2^. Stanley and Bloxham^3,4^ performed numerical dynamo simulations using model geometries to explain Uranus’ and Neptune’s anomalous fields—finding their non-dipolar, non-symmetric magnetic fields are generated by a combination of electromagnetic stresses perturbing the convecting ionic fluid which surrounds a layered, stratified interior. Knowing the phases and properties of H_2_O at the pressure–temperature (*P–T*) conditions of ice giant interiors on their isentropes is critical for validating dynamo simulations—but they are not well understood.

Convection of electrically conducting fluids generates magnetic fields in planetary interiors. If dissociation of molecules occurs in water-rich planets, then total conductivity is comprised of an electronic and ionic contribution. Ionic conduction is caused by the movement of negatively or positively charged ions and in the case of high-pressure H_2_O ice, protonic conductivity properties are crucial to constrain planetary dynamo processes (e.g., Refs.^5,6^). The existence of a proton fluid and an oxygen sub-lattice in the superionic phase raises questions about the response of this phase to electromagnetic stress through protonic fluid motion. Theoretical work has suggested a body-centered cubic (BCC)^7,8^, face-centered cubic (FCC)^9–11^, or orthorhombic (e.g., Ref.^12^) structure of H_2_O is stable at hundreds of GPa pressures and several thousand Kelvin, with bonding and transport properties consistent with a superionic phase.

More recent work^13,14^ use optical reflectivity, absorption measurements, and X-ray diffraction (XRD) to demonstrate the low electronic conductivity of ice and provide experimental evidence for superionic conduction of water ice in an FCC crystal structure, stable at pressure (*P),* temperature *(T)* conditions of ~ 160 GPa and calculated 3000 K. The insulating solid ice phase at comparable pressure and below 2000 K, ice X, is known to have a BCC lattice structure, but does not have superionic properties. In contrast, we find the first XRD evidence for a BCC structure between ~ 100–200 GPa using compression-based solidification of liquid water calculated temperatures up to ~ 5000 K. This BCC crystal structure phase at these *P–T* conditions represents a new phase of ice: ice XIX. Our constraints on the phase diagram of water ice near the conditions of the isentropes of ice giants, like Neptune and Uranus, have implications for their dynamos generating magnetic fields.

## Results

**Figure 1:**
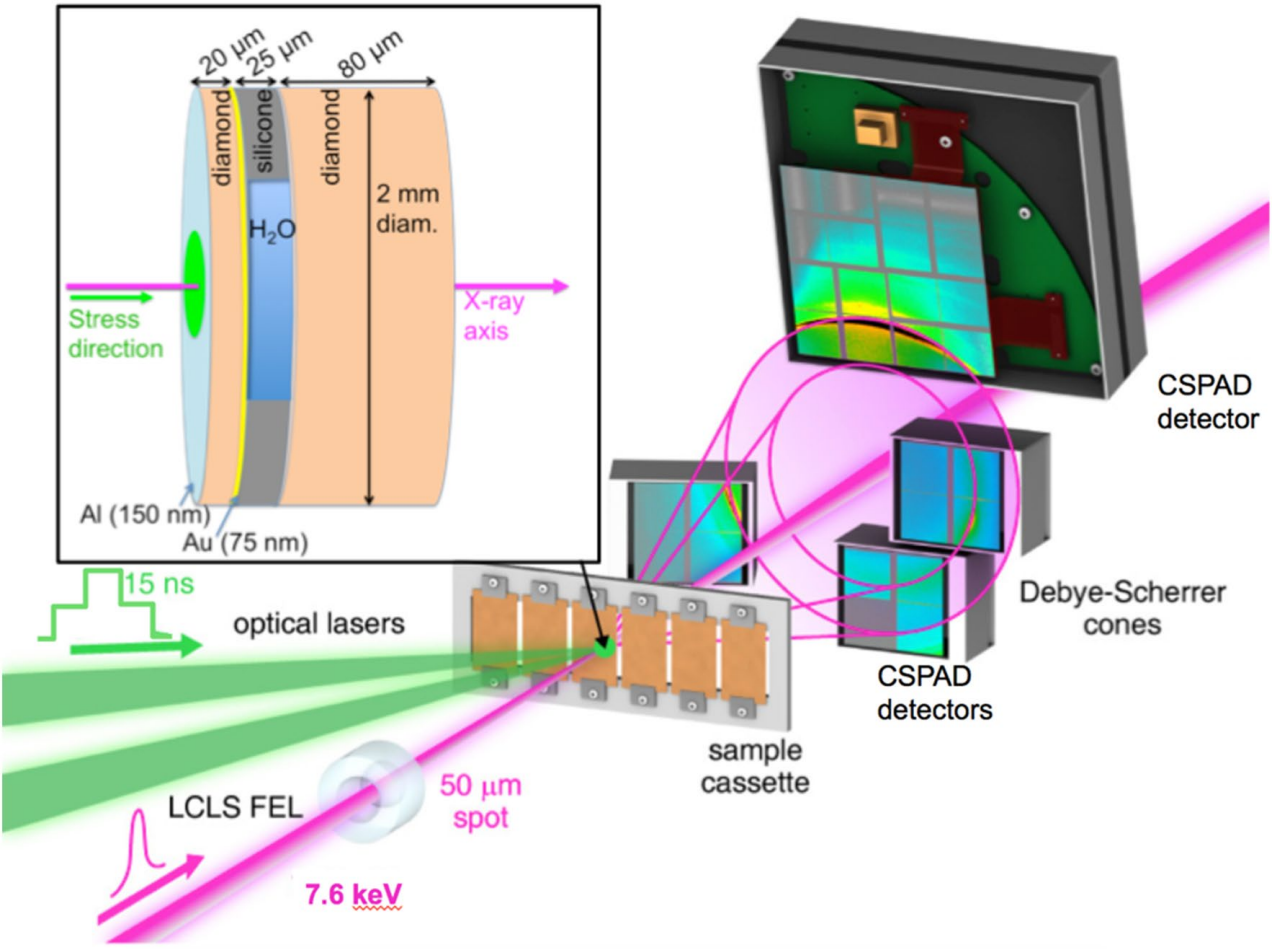
Experimental configuration of the XFEL probe and optical laser pump. The shock solidification behavior of water is captured in a Debye–Scherrer geometry. *Inset:* Schematic of target package with cut away side-view of the water layer.

Simultaneous, in situ XRD and velocimetry data combined with post-shot simulation work was used to examine the lattice structure, pressure, and temperature of H_2_O. Here, atomic structure measurements of compressed liquid water (*ρ*_0_ = 1.0 g/cc; *T*_0_ = 288 K) were made using transmission in situ XRD with 7.6 keV X-rays from the X-ray Free Electron Laser (XFEL) at the Matter in Extreme Conditions (MEC) end-station of the Linac Coherent Light Source (LCLS), SLAC National Accelerator Laboratory (Fig. 1). The applied loading scheme was reverberation compression—achieved through a temporally step-shaped drive laser (see “Methods”). The peak pressure was varied by changing the total number of Joules delivered to the target with a waveplate optic for the long pulse laser. The target geometry consisted of a clamp-style water containment approach [15). Individual packages of sandwiched diamond–water–diamond served as the targets: [20 μm thick chemical vapor deposited (CVD) diamond ablator] + [25 μm deionized water (18 MOhm) layer set by a circular silicone washer (Silastic J, Dow Corning)] + [80 μm CVD diamond window]. Due to the impedance mismatch between the diamond ablator and the water, there is a many step compression sequence as elastic and plastic waves followed by reflections of those waves at interfaces effectively ‘ringing’ up the pressure in the water layer. A 75 nm gold layer was coated on the diamond ‘ablator’ surface in contact with the water sample, serving as the reflective layer for velocimetry and as an internal pressure calibrant to monitor compression via peak shifts in the XRD. The velocimetry data were recorded on a Velocity Interferometer System for Any Reflector (VISAR) diagnostic, simultaneously with the XRD (see “Methods”) to provide an additional pressure constraint. The diamond ‘window’ served as containment for the water and was optically transparent to the VISAR probe allowing velocimetry measurements recording the motion from the Au.

**Figure 2:**
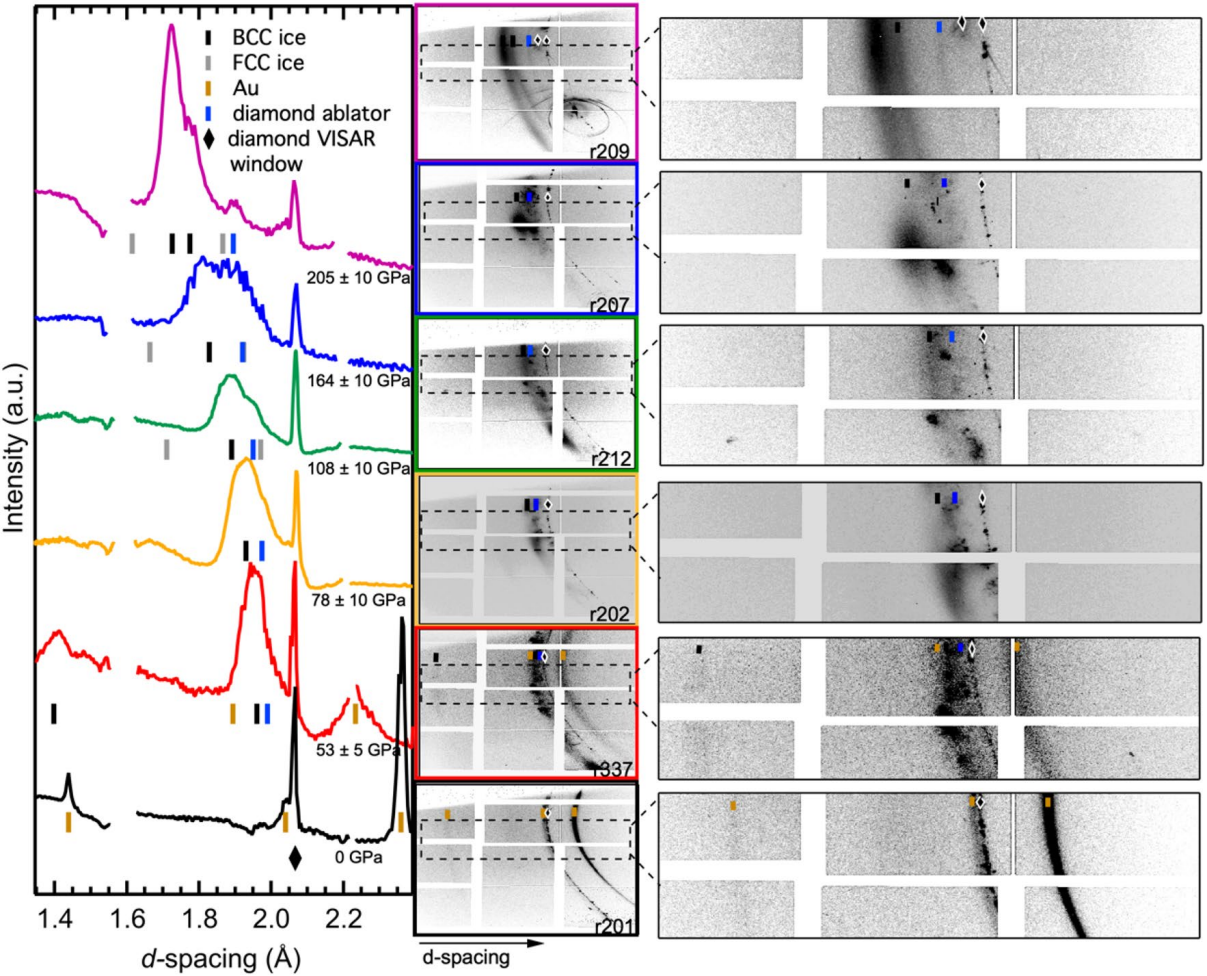
X-ray diffraction data with increasing pressure. Raw integrated traces from all high-pressure water shots without any normalization measured at ~ 7 ns for the highest pressures. Colored tick marks indicate fitted peak center for labeled phase. FCC ice positions are shown for reference to note where peaks would be predicted to appear, but are not observed, at these pressures. The sharp peak at 2.07 Å is the diamond peak (111) at near ambient conditions, labeled (filled diamond) from the VISAR-side window. r337 peak assignment is particularly complicated due to superposed peaks from diamonds and ice. However, due to the presence of the (200) ice peak near 1.4 Å we can constrain the ice (110) to be a component of the diffraction intensity seen at ~ 1.95 Å. Breaks between the detector pads are seen at *d-*spacings of 1.6 and 2.2 Å. Examples of 2-dimensional raw CSPAD images with colored ticks to match phases listed in traces are shown to the right.

Diffraction data, recorded on Cornell-SLAC Pixel Array Detectors (CSPADs), are azimuthally integrated (Fig. 2) as a function of *d*-spacing (Å) (see “Methods”). A representative trace of an integrated XRD pattern at ambient conditions shows strong intensity (111), (200) and (220) Au peaks plus CVD diamond peaks (from both the ablator and window). XRD records the reverberation-compressed sample at an XFEL probe timed to capture the diffraction after peak compression was achieved (~ 7–9 ns). Polycrystalline diffraction peak positions are determined from peak fitting using Fityk^16^; Table 1 for a listing of run numbers, XRD peak *d*-spacings, hkl assignments, lattice parameters, densities, and estimated *P–T* conditions.

**Table 1:** Peak assignment and lattice parameters.

Run	Phase	hkl	*d*-spacing (Å)	*a* (Å)	V(Å^3^)	⍴ (g/cc)	Up (km/s)	P_visar (GPa)	P_xrd (GPa)	Average P (GPa) , T (K)
201										
	Au*	111	2.361(1)	4.079	67.87	19.28			0	
		200	2.043(3)	4.079	67.87	19.28			0	
		220	1.437(1)	4.079	67.87	19.28			0	
	diamond-V,ab	111	2.063(1)	3.574	45.63	3.49	0	0	0	0, 300
337										
	Au*	111	2.233(3)	3.868	57.86	22.61			47.0	
	diamond-V	111	2.063(3)	3.574	45.63	3.49				
	diamond-ab^	111	1.989(4)	3.445	40.89	3.90	1.2(1)	57.0	53.0	
	ice_bcc VII	110	1.961(3)	2.782	21.53	2.78				53(5), 1150(250)
	ice_bcc VII	200	1.397(4)	2.782	21.53	2.78				53(5), 1150(250)
202										
	diamond-V	111	2.069(5)	3.574	45.63	3.49				
	diamond-ab^	111	1.975(3)	3.420	40.00	3.99	1.7(1)	84.6	78.1	
	ice_bcc X	110	1.930(5)	2.730	20.35	2.94				78(10), 1800(350)
212										
	diamond-V	111	2.067(3)	3.574	45.63	3.49				
	diamond-ab^	111	1.949(5)	3.377	38.51	4.14	2.3(2)	114.9	107.8	
	ice_bcc XIX	110	1.889(2)	2.680	19.25	3.11				108(10), 2200(350)
207										
	diamond-V	111	2.071(3)	3.574	45.63	3.49				
	diamond-ab^	111	1.920(7)	3.326	36.79	4.34	3.2(3)	171.7	164.0	
	ice_bcc XIX	110	1.828(6)	2.587	17.31	3.46				164(10), 2700(500)
209										
	diamond-V	111	2.064(3)	3.574	45.63	3.49				
	diamond-ab^	111	1.895(3)	3.282	35.35	4.51	4.5(4)	245.5	205.4	
	ice_bcc XIX	110	1.770(3)	2.504	15.70	3.81				205(10), 5500(500)
	ice_bcc XIX	110	1.725(3)	2.440	14.53	4.12				205(10), 3300(500)
399										
	diamond-V	111	2.069(3)	3.574	45.63	3.49				
	diamond-ab^	111	1.894(4)	3.281	35.32	4.52	4.5(5)	245.5	207.5	
	ice_bcc XIX	110	1.769(5)	2.502	15.66	3.82				207(10), 5500(500)
	ice_bcc XIX	110	1.724(4)	2.438	14.49	4.13				207(10), 3300(500)

*V* VISAR-side diamond, *ab* ablator-side diamond.

*Pressure and temperature determined using Fei et al.^18^ and Marsh^19^.

^Pressure determined using P-rho of McWilliams et al.^20^; Knudson et al.^21^ and Marsh^19^ and LEOS9061^30^.

Diffraction from the (111) peak of the downstream diamond window shows little or no shift from the ambient *d*-spacing of 2.063 Å, indicating that the majority of the window volume is uncompressed. The lowest pressure XRD pattern records the Au (111) and (220) peaks shift to smaller *d*-spacing and broadening – providing a thermally corrected pressure of 47 ± 3 GPa, 1150 K (Ref.^17,18^); Fig. 2, red trace. The ablator CVD (111) diamond peak is also resolvable and shifts, consistent with 53 ± 5 GPa compression^18–20^. Two new XRD features are seen at 1.961(3) Å and 1.397(4) Å corresponding to the BCC ice VII structure, for (110) and (200), respectively, with a density of 2.78 g/cc, as expected in this regime^21^. Within uncertainty, the densities of Au, diamond, and ice are all consistent with a pressure of 53 ± 5 GPa, also in agreement with the VISAR measurement of 56 ± 4 GPa. Using previously published reverberation-compression-based equations of state (EoS) for ice VII^7,19–25^, we estimate a temperature of 1150 ± 250 K for this density.

Upon increasing compression, the Au peaks are no longer resolvable in the XRD due to possible drive light leakage generating thermal expansion peak broadening. Although we clearly see Au peaks in the ambient patterns, above ~ 50 GPa we can no longer resolve the Au. Loss of clarity in the XRD data is likely due to peak broadening to the point that the diffuse scatter intensity distribution across the CSPAD prevents it from being resolved as discrete peaks. Possible reasons for broadening include: (1) reaching temperatures above the melting point of Au at these pressures, and/or (2) thermal expansion due to drive light leakage through the diamond ablator reaching Au layer before the compression process can take place. It has been documented that the drive laser spatial profile can spill over the chamfered drive side of the target mount and damaging neighboring targets. Due to laser light from a preceding shot reaching an adjacent target, the Al flash coating (150 nm) on the drive side of the neighbor target can be damaged. The purpose of an Al flash coating is to prevent drive light from leaking through the ablator. However, if that Al coating was damaged, drive light can reach the Au layer, resulting in premature thermal expansion such that we cannot resolve the peaks.

Peak shifts in the compressed diamond ablator are resolvable and, using these diffraction peaks as a pressure calibrant in combination with the velocimetry record, we track pressure increasing to just over ~ 200 GPa^18–20^. The reported pressures are determined from the XRD of the diamond-ablator and are corroborated, within the uncertainty, by the pressure determined from the velocimetry traces. Pressure uncertainty is taken from the goodness-of-fit value for a Gaussian peak profile of the ablator diamond diffraction peak *d*-spacing, converted to a density uncertainty and used to estimate the and pressure uncertainty with an equation of state. We see the water ice diffraction peak shift from 1.961(3) Å (at 53 GPa) to 1.725(3) Å at the highest pressure. If we continue to assign this feature as a BCC (110) peak, the ice pressure estimates (via EoS from Refs.^7,19–21^) track well with the compressed diamond ablator estimates up to ~ 160 GPa. Beyond this pressure there are discrepancies in the EoS results between quantum molecular dynamics simulations (e.g., Ref.^24^) and previous experiments (e.g., Refs.^21,22,26^) for water. At the highest pressure, 205 ± 10 GPa, indexing the new peak as a BCC (110) gives a lattice parameter of *a* = 2.440 Å, corresponding to a density of 4.12 g/cc. Unfortunately, the (200) peak for the BCC ice structure falls off the detector *d*-spacing range above ~ 75 GPa.

**Figure 3:**
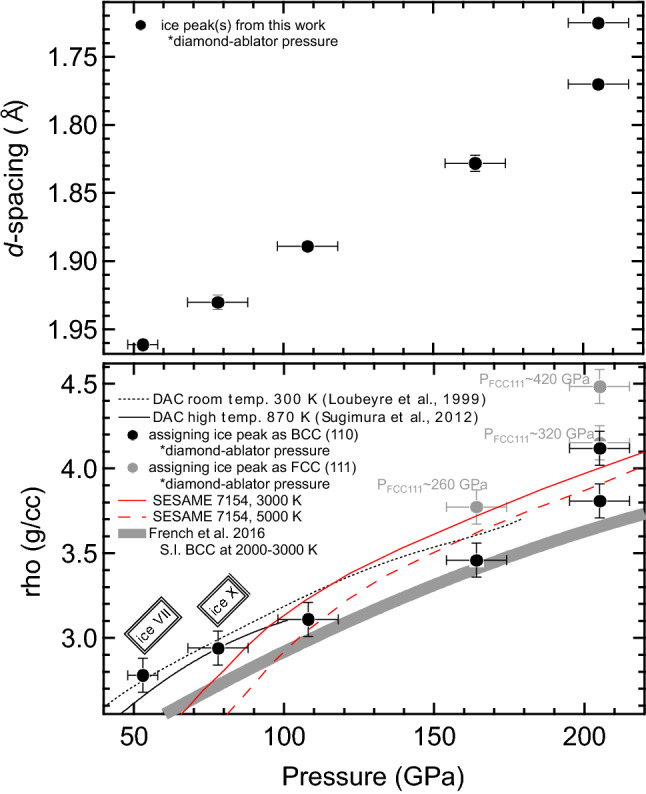
Comparison of *d*-spacing and density for different structures. Measured *d*-spacings for each ice peak plotted with the pressures derived from the diamond-ablator XRD signal (top graph). Black circles (bottom graph) show density trend with pressure assigning this peak as the BCC (110). Dotted black line is the Vinet fit to room temperature, static compression DAC work Loubeyre et al.^40^. Solid black line is the trend for high temperature DAC data Sugimura et al.^35^. We note a reasonable similarity with this trend and the DFT prediction from French et al. (Ref.^25^; solid grey line) for a superionic BCC structure of ice below 200 GPa, but above there is some discrepancy. Red curves are from SESAME 7154 (Ref.^26^) for 3000 K and 5000 K, solid and dashed, respectively. Assigning the same *d*-spacing peak to FCC (111) (grey circles) shows a marked jump in density using the diamond-ablator based pressure which does not fit any predicted trend, nor do we have the corresponding FCC (200) peak which would be in the detector range. If we use French et al.^25^ EoS for superionic FCC ice to determine pressure of the ice, there would be a marked jump in pressure which does not corroborate the diamond-ablator pressure, or velocimetry-based pressure or laser drive intensity-pressure calibration data.

**Figure 4:**
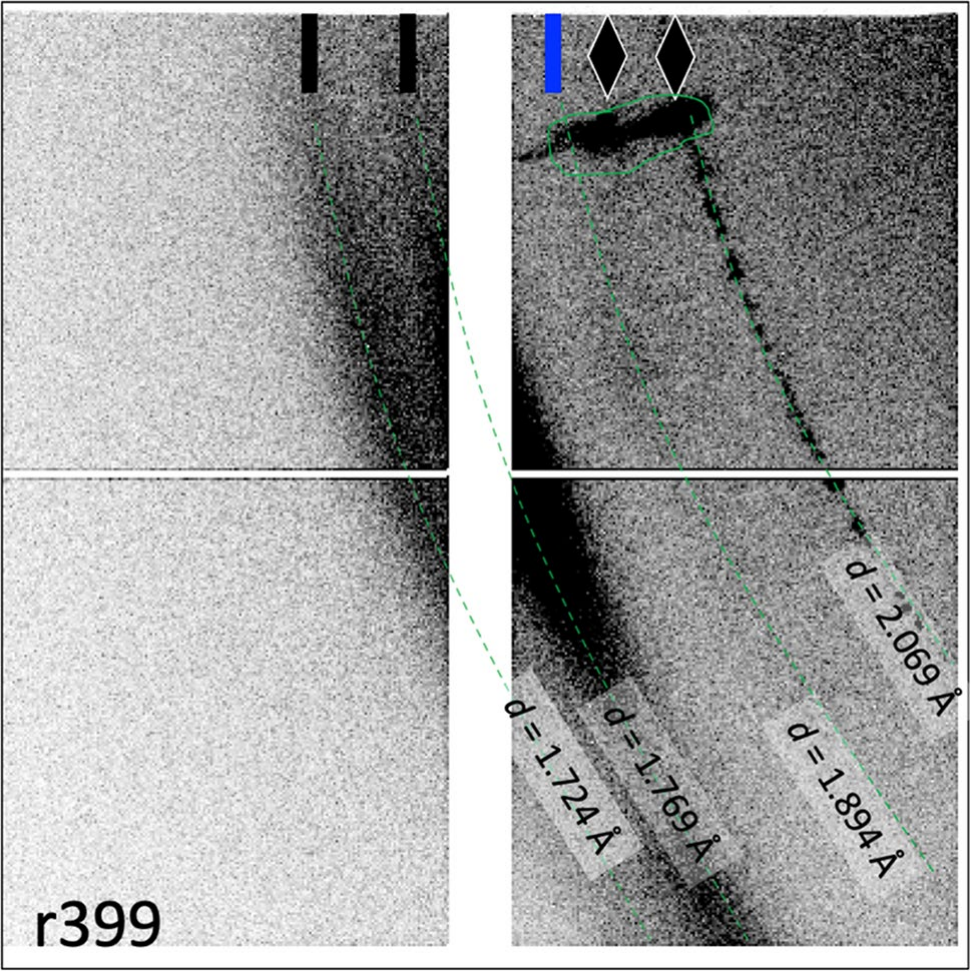
Raw data from Run 399. Repeat of conditions from Run 209, this Run 399 shows diffraction signal from ice XIX with the same doublet feature (black ticks, *d*-spacings = 1.724 Å, 1.769 Å), the ablator diamond (blue tick, *d*-spacing = 1.894 Å), the VISAR window diamond (black diamonds, *d*-spacing = 2.069 Å and elongated feature at the top of the image which could be due to a small volume fraction of the single crystal responding to the stress distribution from compressive wave interactions). Light green lines are guides for the eye or to outline features.

Above the ice VII and ice X *P–T* stability fields, we can test the viability of the FCC, hexagonally-close packed (HCP), and orthorhombic structures, assuming the geometric constraints of packing efficiency or close packing oxygen in three dimensions (e.g., Refs.^10,12^). Our procedure was to test assignment of the new peak visible in pressures above ~ 150 GPa to an FCC, HCP, or orthorhombic (*Pbcm*) structure and then inspect the 2-dimensional CSPAD images for any diffraction intensity located near a predicted (hkl) *d*-spacing position for that structure. If we assign the FCC (111) peak to the 1.828 Å feature, the corresponding FCC (200) should be at 1.583 Å which should then shift to 1.50 Å with compression. We do not see any XRD signal at these positions on the CSPADs. Similarly, if the HCP structure were assigned at comparable ice densities, the (101) is missing at its predicted 1.843 Å, or 1.678 Å, respectively for *ρ*_HCP_ = 3 and 4 g/cc. Regarding the orthorhombic *Pbcm* structure—we also check for peaks using a linear extrapolation of lattice parameters, e.g., Ref.^12^, to ~ 200 GPa to look for (110) and (101) peaks at 1.96 Å and 1.47 Å, respectively. Figure 3 compares *d*-spacing vs pressure and corresponding densities at each pressure assuming a BCC or FCC structure, including previous diamond-anvil cell data. Due to the absence of any corresponding FCC, HCP, or *Pbcm* peaks, each expected to be within the detector *d*-spacing coverage with predicted relative intensities of these peaks well above the noise floor of the detectors, and a BCC ice density consistent with velocimetry-based pressure estimates and compressed diamond-ablator pressures estimates, we conclude that the ice structure seen in the XRD data is BCC. At similar shot conditions, the formation of this BCC structure is reproducible (Fig. 4).

Previous dynamic compression induced disorder-order transitions (e.g., Ref.^27^) have reported randomly-oriented nanocrystalline growth of the high pressure phase as seen in the uniformity of Debye–Scherrer ring intensity and the relative peak intensities matching a randomly orientated powder distribution. However, we see interesting trends in the change in powder XRD texture for both the ice phase and diamond above 100 GPa. The CVD ablator diamond signal remains spotty, showing a similarly sized grain structure as it compresses in the elastic regime up to the ~ 80 GPa HEL. Above this pressure, we see a gradual increase in ring smoothness and peak broadening up to the highest pressure of ~ 200 GPa where the intensity is more uniform over the azimuthal range available. This may indicate that the grain size is likely decreasing and orientations are becoming more random. The ice VII and ice X diffraction are large, broad and spotty up to over 100 GPa. Then as the ice X transitions to ice XIX above ~ 150 GPa we see these larger spots become more diffuse at the edges, perhaps indicating some increase mosaicity and/or crystallites with preferred orientation with respect to the compression direction. In the highest pressure shot there is an apparent concentration of diffraction intensity for the BCC ice (110) peak near the top of the detector. This is consistent with the horizontally polarized XFEL probe (considering the orientation of the CSPAD active areas with respect to the XFEL propagation direction). Additionally, we note heterogeneous growth of ice crystals on target component interfaces may have a needle-like geometry^15,28^, such that nanocrystallites develop preferential orientation with respect to the X-ray probe direction. This could also contribute to a concentration of diffraction intensity at this location on the detector.

**Figure 5:**
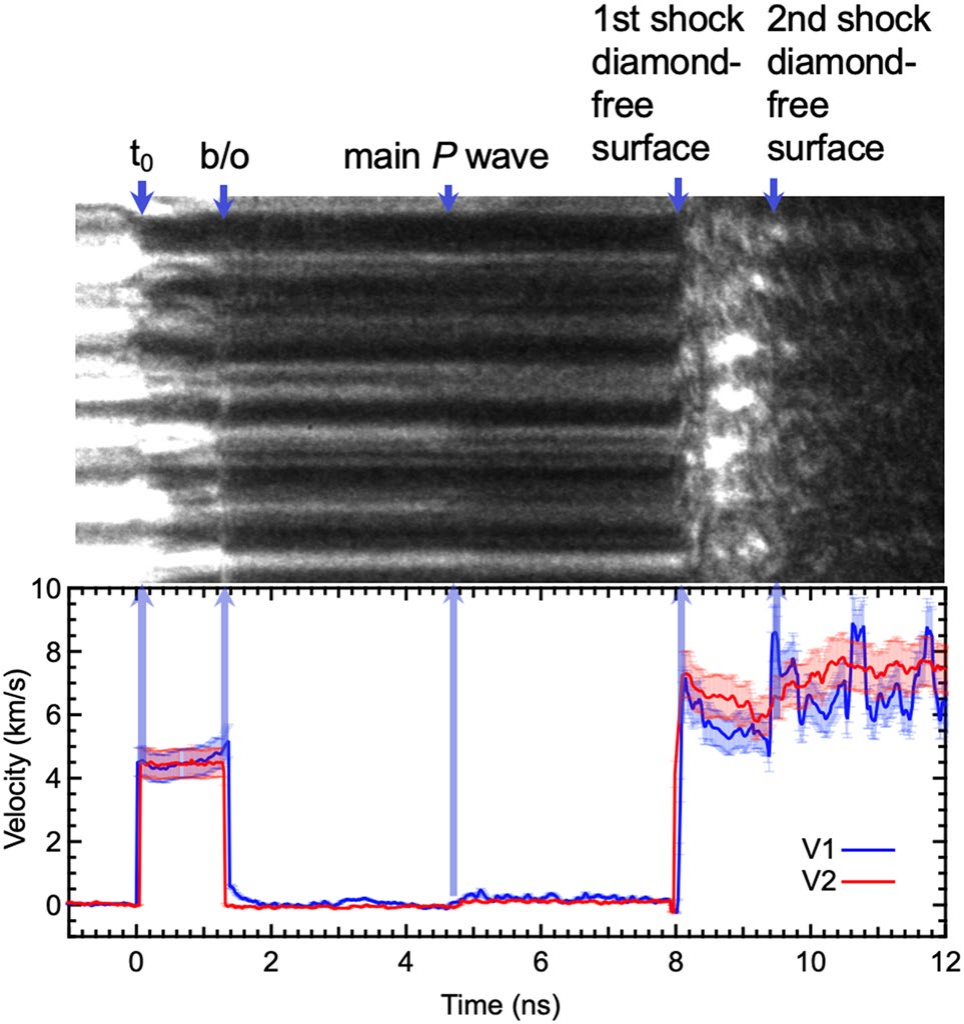
Velocimetry data from reverberation compression of water to ~ 200 GPa. Example raw VISAR1 (blue line & streak camera image) and VISAR2 (red line) data from Run 209 over a 28 μm region showing particle velocity (Up) of the diamond ablator (~ 4.5 km s^−1^) and free surface velocities (Ufs) from the 1st and 2nd shock arrivals at the diamond VISAR window (~ 7–7.5 km s^−1^) ranging from 4.4 to 6 km s^−1^. Due to VISAR quality, the Up uncertainty is ~ 10%. Breakout (b/o) of the diamond elastic wave into the water layer occurs at ~ 1.5 ns, followed by the main pressure wave reaching the VISAR-side diamond-water interface at ~ 4.5 ns. The 1st and 2nd shockwave arrivals reach the VISAR-side diamond-vacuum interface at ~ 8 ns and 9.5 ns, respectively. Drive laser parameters listed in Table 2.

**Figure 6:**
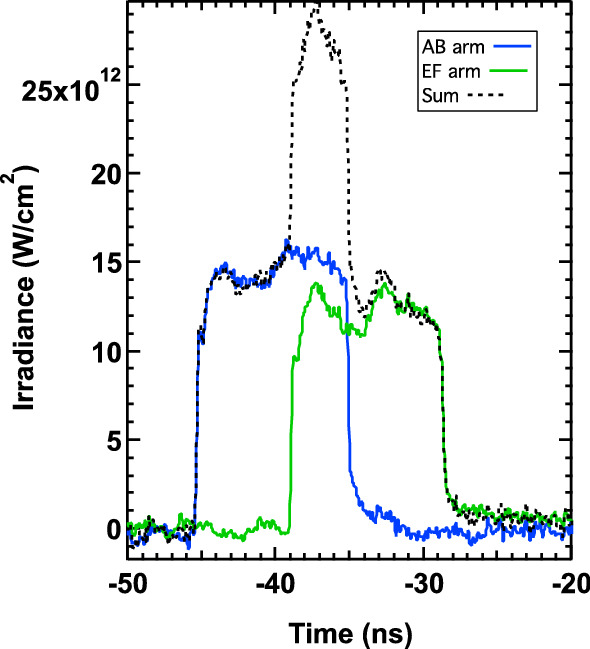
Oscilloscope traces of drive laser temporal profiles. Blue and green traces show the two 10 ns flat-top profiles offset by ~ 5 ns to yield a stepped ~ 15 ns drive pulse profile. Two separate laser ‘arms’ are combined to generate the compression wave, here labeled AB and EF corresponding to names of the capacitor banks. The total irradiance as seen by the target is the sum of the two drive profiles shown by the dotted black line.

Velocimetry data was obtained by analyzing the line VISAR interferograms measured in the experiments with an image reduction routine^29^, which employed a Fourier transform method to extract the interferograms’ phase information. The spatially resolved velocity histories were acquired by applying the experimental velocity-per-fringe to the extracted phase map of the data (representative VISAR, Fig. 5). The drive laser pulse used to achieve these conditions is shown in Fig. 6. Equations of state (EoS) from SESAME 7154 and LEOS 9061 were used for the water and diamond ablator, respectively^26,30^, finding these are comparable to those used in Millot et al.^13,14^. Due to the large impedance mismatch between the diamond and the water, the initial shock wave in diamond generates a release wave at the diamond-water interface which is reflected back into the diamond ablator. The impedance difference persists and sets up a reverberating shock in the diamond ablator. Breakout of the first shock in the ablator diamond into the water is at 1–2 ns (labeled ‘b/o’ for breakout, Fig. 5). The ablator diamond reverberation results in the diamond plastic wave overtaking the weak elastic wave in the water and reached the water-VISAR diamond interface at roughly ~ 5 ns. A 1st and 2nd shock wave transits the VISAR-side diamond to breakout into vacuum by ~ 8 ns and 9.5 ns.

**Figure 7:**
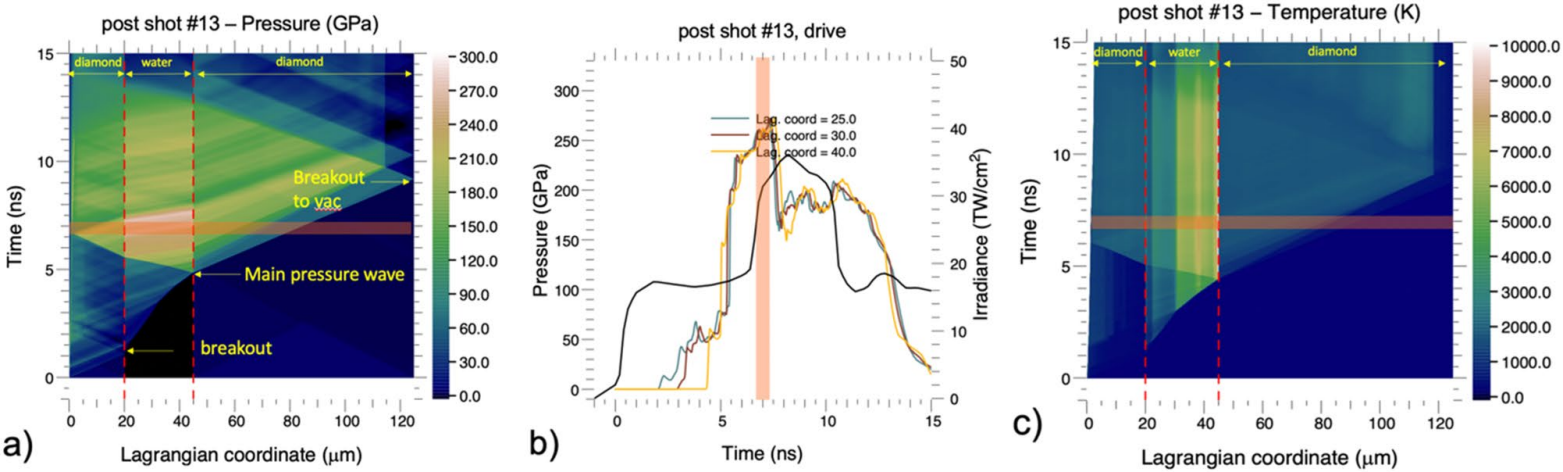
Lagrangian distance-time diagrams. A post-shot simulation for (**a**) pressure labeled by color code (**b**) pressure line outs of specific Lagrangian coordinates in the water layer and (**c**) temperature distribution. In (**a**) and (**c**), the red dashed line shows the water region. Solid black line in (**b**) shows drive profile. Probe time of the X-rays was at 6.95 ± 0.35 ns (orange bar) where line width includes probe time uncertainty derived from rise time of first pulse (0.15 ns) and timing jitter of laser (0.20 ns).

The HYADES Radiation Hydrodynamics code^31^ was used to perform a post-shot simulation and model the wave propagation through the target package (Fig. 7). These simulations compare well with velocimetry data—confirming the timing of expected features, like breakout of the elastic wave from the ablator-diamond, the main pressure wave reaching the downstream water-diamond interface near 4.5 ns, and shock waves breaking out into vacuum at around ~ 8–9 ns. Since we had no separate temperature diagnostic, we can only rely on this post-shot simulation temperature estimate and known *P–T* EoS for water under reverberation compression conditions^21–23,26^ to constrain our temperature. Post-shot simulations were completed for high- and low-pressure shots using the Hyades Radiation Hydrodynamics code. We found that these shots required a multiplier of 0.45 to obtain a best match to VISAR data. A typical multiplier value is ~ 0.7 (Ref.^32^), however our small multiplier value is indicative of extensive laser energy loss before reaching the target. This could be due to optics/coating damage in the beam path decreasing the delivered intensity reaching the target – perhaps up to 65%.

Diffraction from the highest pressure shot shows a doublet feature for the BCC (110). The hydrodynamic simulation shows a bimodal temperature distribution at the 7 ns probe time, present in the ice in two discrete ice layers: at ~ 3300 K and at 5500 K. This ~ 2000 K temperature difference between the layers would manifest as a density difference of 30–35% in the ice, which is consistent with the peak separation using the SESAME 7154 Gruneisen parameter (Ref.^26^). Because of the uncertainty in pressure and temperature conditions for this shot we list out two data points for BCC, one for each peak of the doublet giving: 205 GPa, 3300 K and 205 GPa, 5500 K.

## Discussion

**Figure 8:**
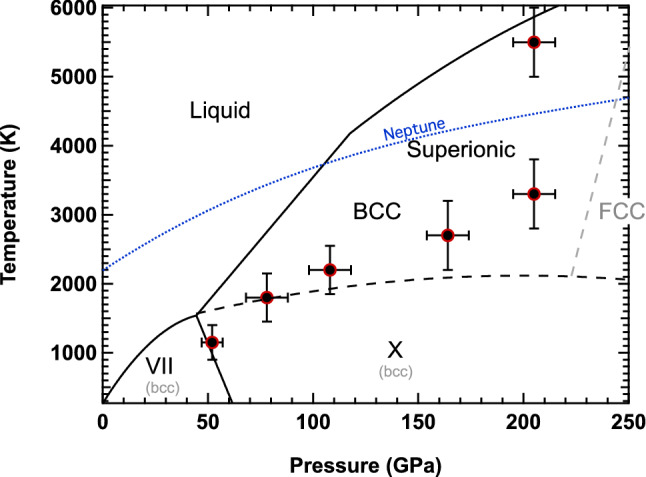
Phase diagram of H_2_O. Revised phase diagram for H_2_O (adapted from Refs.^9,10,14,25,40,41^ including our data for BCC-structured ice (black circles) which shifts the FCC boundary to higher pressures in line with theoretical predictions from Refs.^10,25^). Blue dashed line is the Neptunian isentrope^36^.

Direct observation of the crystal structure of H_2_O ice at a pressure of ~ 200 GPa and a calculated temperature of 5000 K has implications ranging from the fundamental physics and chemistry of H_2_O to ice giant dynamo evolution. XRD presented here provides the first evidence of a BCC ice structure at these conditions. Consistent with superionic behavior, water is predicted to have a band gap (2–3 eV) at these conditions causing it to absorb visible light (e.g., Ref.^7^), and we do see the loss of reflectance of the 532 nm probe light off the Au as the shock front transits the H_2_O layer. However, since our velocimetry records are inconclusive regarding a direct transport property measurement, we also consider alternate explanations. For instance, reflectance loss at ~ 2–8 ns (Fig. 5) could also result from the light scattering off BCC ice grain boundaries (or a combination of both phenomena). Using the Scherrer Equation^33^:$\phi =\frac{K\lambda }{\beta cos\theta }$, where *ϕ* = grain size; *K* = dimensionless shape factor (commonly set to 0.9); *λ* = X-ray wavelength; *β* = line broadening at full width at half maximum (FWHM) minus instrumental broadening (0.03°); *θ* = Bragg angle, the BCC (110) ice peak width gives a grain size of 21 ± 2 nm, similar to the findings of Millot et al.^14^. This small grain size could lead to scattering of the VISAR probe light, which could cause the apparent loss of Au reflectivity. We do note that previous experiments^13,14,34,35^ and computations (e.g., Ref.^9,24,25^) have shown that the *P–T* conditions achieved here is within the superionic phase stability field. Applying our new XRD data to the phase diagram of high pressure ice confirms the BCC structure previously theorized is stable at these conditions (e.g., Refs.^10,14,25^) (Fig. 8) in the superionic regime. Recent pioneering work by Millot et al.^13,14^ has also examined the phase diagram of water, to pressures beyond this paper, under laser-driven shock-compression. We compare the lattice structure of ice at similar *P,T* conditions and find evidence of a BCC ice structure near the liquid boundary suggesting the FCC phase stability region can be pushed out to higher pressure. Millot et al.^14^ find an FCC ice extending to the liquid boundary. Our data suggest pushing this FCC boundary out to higher pressures (> ~ 250 GPa) along the isentrope. However, this pressure assignment, based on diamond XRD could represent the lower bound for the ice pressure, and in fact, be at higher pressure as indicated by hydrocode assessment. Our results are consistent with all but one of the data points reported by Millot et al.^14^. The reason for this discrepancy is not yet understood, but could be related to issues in diffraction quality signal/noise and indicates the need for more experimental investigations working to resolve real-time diffraction for phase with higher *Q*-range, velocimetry for pressure, and pyrometry for temperature determination all collected with in situ diagnostics.

**Figure 9:**
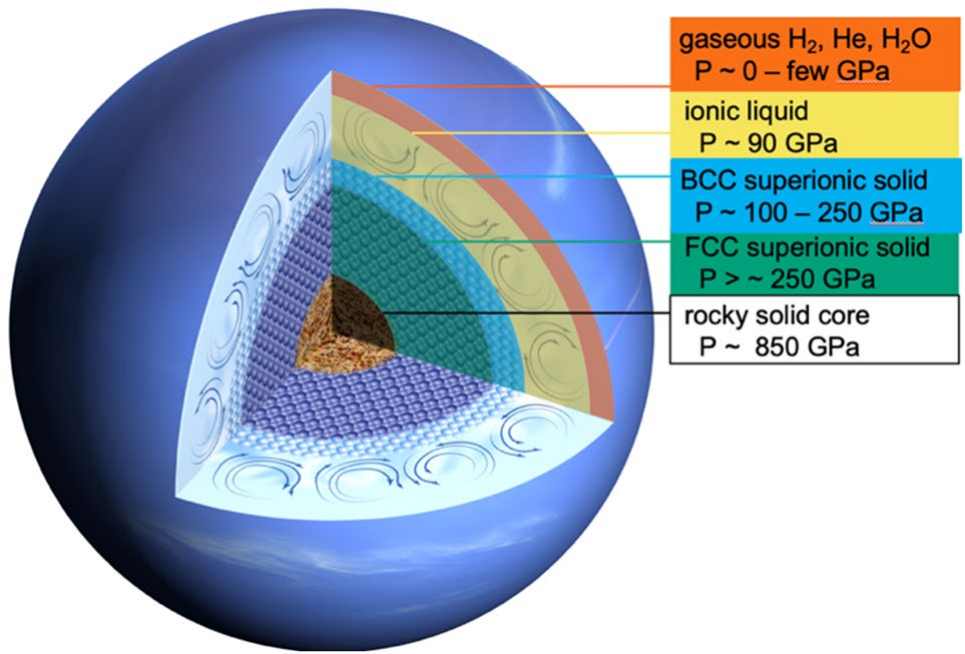
Neptune interior with multiple superionic layers. Ice giant interiors, like Neptune, have different layers of superionic ice. The molecular envelope of He, H_2_ and H_2_O gas is largely insulating and the convecting ionic liquid layer may have ionic conductivities of a few to 100 (Ω cm)^−1^. However, the superionic solid BCC and FCC layers can have comparable protonic conductivity 100 (Ω cm)^−1^ or up to two orders of magnitude larger, depending on the estimated proton mobility (or diffusion coefficient *D*_*H*_ = 1.8e−3 cm^2^s^−1^ (Refs.^6,36^) to 0.5 cm^2^ s^−1^ (Ref.^9^) at relevant pressures and temperatures along the isentrope.

Due to the nature of packing of the oxygen sub-lattice, the BCC structure is generally thought to have a higher hydrogen mobility than the FCC structure. The BCC structure allows the hydrogen atoms to migrate freely between different, connected interstitial sites, i.e., tetrahedral or octahedral, whereas the FCC structure has only one less connected tetrahedral site than BCC^9^. This mobility is commonly assessed in molecular dynamics calculations as the hydrogen diffusion rate^8,9,11,36^. A linear extrapolation of the hydrogen diffusion constant with density to the conditions probed in this study suggests the BCC hydrogen diffusion constant is 40% higher than the FCC diffusion constant^9^. The values for the diffusion coefficient vary over many orders of magnitude, 1.8e−3 cm^2^s^−1^ (Refs.^11,36^) to ~ 0.5 cm^2^s^−1^ (Ref.^9^) for pressures and temperatures measured in this study for a BCC structure, depending on the molecular dynamics simulation parameters. The relationship between hydrogen diffusion coefficient (*D*_*H*_) and protonic conductivity (σ) is governed by the Nernst-Einstein equation^37,38^: $\sigma =\frac{{fnD}_{H}{q}^{2}}{RT}$, where *f* is non-dimensional geometrical constant taken to be 1, *n* is the molar concentration per unit cell volume, *q* is the charge, *R* is the gas constant, and *T* is the temperature, and shows a proportional relationship between hydrogen diffusion coefficient and protonic conductivity. For our BCC lattice parameter (2.440 Å), and a *D*_*H*_ of 1.8e−3 cm^2^s^−1^ (Ref.^36^) we find a protonic conductivity of 102 (Ω cm)^−1^, whereas an FCC structure at the same pressure would be ~ 20% lower conductivity. This is in contrast to the liquid outer region of Neptune where ionic conductivity at 25 to 100 GPa, ranges from < 1 to 30–90 (Ω cm)^−1^, respectively (e.g., Ref.^6,39^). Confirmation of the extension of the BCC high-pressure stability field in the superionic regime results in higher than predicted protonic conductivity to greater depths in ice giant interiors (Fig. 9) which can respond well to magnetic stress.

Ice giant dynamos are generated in a convecting, fluid layer of electrically conductive water, ammonia, and methane. Solid ice layers cannot participate in the dynamo action through fluid motions. However, dynamo complexities arising from layered superionic ices with high but differing proton mobilities will influence the magnetic field properties. Here we have shown that solid ice is stable to over 200 GPa and 4400 K in a BCC structure. Combined with previous measurements that indicate that water ice is superionic in this *P–T* region^13^, this suggests that the lower boundary of the dynamo-generation region is likely related to the location of the superionic phase change, especially if that phase change results in a solid ice layer (e.g., Ref.^6^), as seen here. Our data change the sub-liquid layer from FCC superionic solid ice to BCC superionic solid ice, resulting in a 40% increase in protonic conductivity. The magnetic fields generated in the ionic liquid layer will interact differently with a BCC ice layer than they would an FCC ice layer due to this difference in conductivity. For example, the magnetic fields generated in the ionic fluid layer are time-varying and the skin depth of penetration of magnetic fields into any solid sub-layer depends on the conductivity of that solid material. A higher conductivity solid sub-layer would preferentially repel magnetic fields, limiting their length scales. Although dynamo simulations have been able to produce multipolar magnetic fields without an enhanced conductivity of a solid interior (e.g., Ref.^3^), the smaller length-scales resulting from the enhanced repulsion of a higher conducting solid interior would promote the generation of multipolar magnetic fields—consistent with measurements made by Voyager II for Uranus and Neptune.

## Methods

### Experimental design

Quasi-monochromatic (dE/E = 0.2–0.5%), fully transverse coherent, 7.603(30) keV x-ray pulses of 40 fs duration with an average of ~ 10^12^ photons per pulse, were incident over a 50 μm diameter spot on the target package. An X-ray only shot was collected before the drive shot as a reference. The 50 μm XFEL beam spot did not produce any observable x-ray damage to the target. Metal coatings on the diamond ablator served to absorb the drive laser (150 nm Al on upstream side) and act as the reflective layer for velocimetry measurements (75 nm Au on downstream side).

The optical drive laser was defocused to a 100 μm diameter spot at FWHM with a Gaussian spatial profile to achieve focal spot intensity of ~ 10^13^ W cm^−2^ (Table 2). The angle between drive laser arms and XFEL probe was 22°. An ablation-driven compression wave was launched parallel to the sample normal over a 15.0 ns profile from a frequency doubled Nd:Glass laser system (λ = 527 nm). By adjusting a waveplate optic on the long pulse laser, we could increase/decrease the total number of Joules in the drive pulse and achieve a range of pressures. The applied loading scheme is reverberation compression and was designed to achieve peak pressure and temperature in the water layer near the 7–9 ns X-ray probe time. The temporal drive profile was achieved by temporally advancing one of two laser beams. The first pulse, 10 ns duration, characterized by a ~ 1.6 × 10^13^ W cm^−2^ intensity, pseudo flat-top profile (Fig. 6, blue curve designated the AB Arm) was followed by a second pulse, after 5 ns. This second pulse was slightly less intense, 10 ns duration at 1.4 × 10^13^ W cm^−2^ intensity, pseudo flat-top profile (Fig. 6, green curve designated EF Arm). The target was exposed to the sum of these pulses in time—looking like a step shape in irradiance after 5 ns. The optical laser and X-ray beam were spatially overlapped and operated in single shot mode. The absolute time zero corresponds to overlap of their leading edges. For each shot, a time delay was selected for the XFEL pulse relative to the optical laser pulse with a jitter of 0.35 ns. XRD pattern was captured by CSPADs constructed of individual application-specific integrated circuits (ASICs).

**Table 2:** Drive laser parameters.

Run	Radius [cm]		Energy [J]	Duration [ns]	Spot size [cm^2^]
	r_a	r_b			
r209AB	0.0048	0.0063	15.3	10.2	9.50018E−05
r209EF	0.0053	0.0058	13.4	10.2	9.65726E−05

Drive laser spatial profile (radius) was made using an equivalent plane monitor at the target position and captured as a CCD tif image. ImageJ was use to extract the illumination profile and FWHM from this image. Energies are calibrated using a diode recording light leakage from a mirror in the laser enclosure. Pulse duration is recorded on an oscilloscope for each shot.

The VISAR diagnostic resolves the velocity histories determined from a phase map of the data. Etalon thicknesses of 25.001 mm and 11.006 mm for Mach–Zehnder interferometer beds 1 and 2, respectively, enable a comparison of the two different velocity–time profiles. The profile match, and unique Up determination, is obtained from the correct number of 2π fringe jumps allowed by the etalons. The target package (diamond–water–diamond) combined with this temporal drive profile were designed to generate the following sequence of events. First, the AB arm is incident on the diamond ablator and begins the ablation process setting up a shock wave in the ablator diamond. The impedance mismatch sets up a reverberating shock in the diamond ablator. Then, a weak shock traverses the water to ~ 25 GPa on the principal Hugoniot as transmitted by the elastic wave in diamond. The diamond plastic wave overtakes the weak elastic wave in the water and is the main compressive wave in the water reaching the diamond VISAR window at ~ 5 ns. This plastic wave sends a reverberation wave back into the water/ice again due to impedance mismatch. At 5 ns, the second laser fires, EF arm, attempting to support continued reverberations.
